# Benzodiazepine Use and the Risk of Dementia in the Elderly Population: An Umbrella Review of Meta-Analyses

**DOI:** 10.3390/jpm13101485

**Published:** 2023-10-12

**Authors:** Chieh-Chen Wu, Mao-Hung Liao, Chun-Hsien Su, Tahmina Nasrin Poly, Ming-Chin Lin

**Affiliations:** 1 Department of Exercise and Health Promotion, College of Kinesiology and Health, Chinese Culture University, Taipei 111396, Taiwan; drluiswu@gmail.com (C.-C.W.); chsu@ulive.pccu.edu.tw (C.-H.S.); 2 Department of Healthcare Information and Management, School of Health Technology, Ming Chuan University, Taipei 111, Taiwan; 3 Superintendent Office, Yonghe Cardinal Tien Hospital, New Taipei City 23148, Taiwan; mhl5488@gmail.com; 4 Department of Healthcare Administration, Asia Eastern University of Science and Technology, Banciao District, New Taipei City 220303, Taiwan; 5 Graduate Institute of Sport Coaching Science, College of Kinesiology and Health, Chinese Culture University, Taipei 111396, Taiwan; 6 Graduate Institute of Biomedical Informatics, College of Medical Science and Technology, Taipei Medical University, Taipei 110, Taiwan; d610108004@tmu.edu.tw; 7 International Center for Health Information Technology, College of Medical Science and Technology, Taipei Medical University, Taipei 110, Taiwan; 8 Department of Neurosurgery, Shuang Ho Hospital, Taipei Medical University, New Taipei City 235041, Taiwan

**Keywords:** benzodiazepine, dementia, Alzheimer’s disease, meta-analysis

## Abstract

The prevalence of dementia among the elderly is high, and it is the leading cause of death globally. However, the relationship between benzodiazepine use and dementia risk has produced inconsistent results, necessitating an updated review of the evidence. To address this, we conducted an umbrella review of meta-analyses to summarize the available evidence on the association between benzodiazepine use and dementia risk and evaluate its credibility. We systematically evaluated the meta-analyses of observational studies that examined the connection between benzodiazepine use and dementia risk. For each meta-analysis, we collected the overall effect size, heterogeneity, risk of bias, and year of the most recent article and graded the evidence based on pre-specified criteria. We also used AMSTAR, a measurement tool to evaluate systematic reviews, to assess the methodological quality of each study. Our review included five meta-analyses encompassing 30 studies, and the effect size of the association between benzodiazepine use and dementia risk ranged from 1.38 to 1.78. Nonetheless, the evidence supporting this relationship was weak, and the methodological quality of the studies included was low. In conclusion, our findings revealed limited evidence of a link between benzodiazepine use and dementia risk, and more research is required to determine a causal connection. Physicians should only prescribe benzodiazepine for appropriate indications.

## 1. Introduction

Dementia is the most common disease among the elderly population and is the leading cause of mortality worldwide [1]. The incidence of dementia has increased significantly with the increasing trends of the aging population [2,3]. Currently, over 55 million individuals are living with dementia, a number that is expected to grow to 78 million in 2030 and 139 million in 2050 [4,5]. As there is no treatment available to cure dementia, it has appeared as a major public health and economic issue. Identifying potential risk factors for dementia is complex and challenging. Nonetheless, age, depression, diabetes, smoking, air pollution, and head trauma have emerged as significant risk factors for dementia [6,7,8].

Benzodiazepine is a widely used medication to manage anxiety, depression, and insomnia [9]. Multiple studies indicate that benzodiazepine use is associated with numerous severe adverse events, including falls [10], hip fractures [11], and mortality [12]. Recent epidemiological studies have reported an elevated risk of dementia among patients using benzodiazepines [13,14]. However, a comprehensive quantitative analysis of the available evidence concerning dementia risk among benzodiazepine users is lacking. Despite the numerous studies that have investigated the connection between benzodiazepine and dementia risk [15,16,17], including systematic reviews and meta-analyses [18,19,20], a detailed analysis is still unavailable.

To our best knowledge, no comprehensive attempt has been made to compile and evaluate the available body of evidence. As a result, we conducted an umbrella review of meta-analyses to provide a comprehensive overview of the reported connection between benzodiazepine and dementia risk. The assessment of methodological quality, identification of potential bias, and presentation of up-to-date evidence would help in the correct interpretation of the existing data and inform clinical and policy decisions.

## 2. Methods

### 2.1. Study Protocol

An umbrella review of meta-analyses and systematic reviews reporting on the associations between benzodiazepine use and the risk of dementia was performed according to the Preferred Reporting Items for Systematic Reviews and Meta-Analyses (PRISMA) guidelines [21,22]. This study was not registered in PROSPERO.

### 2.2. Search Strategy

Our search strategy involved using electronic databases such as PubMed, Scopus, and Web of Science to find relevant articles in English published between January 1990 and January 2023. We used the following search terms: ‘(benzodiazepine) AND (dementia OR Alzheimer’s disease) AND (meta-analysis OR systematic review)’. Our search included meta-analyses of randomized controlled trials (RCTs) or observational studies. Articles were reviewed by screening the titles, abstracts, and full texts to decide which ones to include or exclude. We also searched the references of retrieved articles for relevant meta-analyses but did not find any additional ones.

### 2.3. Eligibility Criteria

To be eligible for inclusion in our study, articles had to be meta-analyses or systematic reviews of both RCTs and observational studies reporting on the association between benzodiazepine use and the risk of dementia. We excluded review or systematic review articles without meta-analysis, in vitro studies, and genetic studies. Additionally, we excluded meta-analyses that lacked the necessary data to perform a reanalysis.

### 2.4. Data Extraction

Two authors (MMI and TNP) independently extracted the data, and any discrepancy during the data extraction process was resolved through discussion. The following data were extracted from eligible meta-analyses: first author, year of publication, the study design, the number of included studies, the number of dementia patients and total participants, and overall effect size with a 95% confidence interval (CI), *p*-value, and I^2^ value.

### 2.5. Quality Assessment

The Measurement tool to Assess Systematic Reviews (AMSTAR-2) was used to assess the quality of the included meta-analysis. AMSTAR-2 consists of 16 items to assess the quality and it is recommended by Shea et al. [23]. Each item is categorized on a three-option scale and 7 items are defined as critical to ascertain the quality of meta-analyses. The quality of the study is considered critically low quality if it fails to achieve more than one of these critical items, high quality if none or just one non-critical item is not addressed. Any confusion in assigning quality ratings was resolved either by discussion between the same two reviewers or by discussing with a third reviewer.

### 2.6. Determining the Level of Evidence

The level of evidence of each meta-analysis was classified into four different levels of strength of the evidence of the association between benzodiazepine use and the risk of dementia. The criteria of level of the evidence were based on effect size, *p*-value, and heterogeneity between the studies [24]. The criteria were as follows:

Convincing evidence: Evidence of the association was considered convincing if the effect size was large, calculated using a random-effect model, and *p* < 0.001. Moreover, no heterogeneity was observed between studies.

Suggestive evidence: Evidence of the association was considered suggestive if the effect size was large, calculated using a random-effect model, and *p* < 0.05. Moreover, no heterogeneity was observed between studies.

Weak evidence: Evidence was considered weak if the study effect size was small, calculated using a random-effect model, and *p* < 0.05. Moreover, large heterogeneity was found between studies.

Non-significant association: If the reported effect size was not statistically significant (*p* > 0.05).

## 3. Results

### 3.1. Literature Review and Study Characteristics

Electronic databases search yielded 15 systematic review and meta-analysis. Initially 10 studies were removed due to duplication. Moreover 3 studies were excluded by screening of titles and abstracts. Finally, 5 studies were selected in this umbrella review [9,25,26,27,28] (Supplementary Figure S1).

### 3.2. Description of Included Studies

Table 1 shows the basic characteristics of included studies. All the meta-analysis published between 2015 and 2019. It covers a total of eighteen years publications (2002–2018). All the included studies searched articles in PubMed, EMBASE, and evaluated the risk of bias using Begg’s and Egger’s test. Only one study found significant publication bias and only one study reported that benzodiazepine use was not associated with an increased risk of dementia due to protopathic bias. Sixteen studies were included in these five meta-analyses and studies characteristics are given in the supplementary file.

### 3.3. The Effect of Benzodiazepine on Dementia Risk

The findings of each meta-analysis on the effect of benzodiazepines on dementia risk are summarized in Table 2. All included meta-analyses showed a significant risk of dementia among benzodiazepine users (all studies had weak evidence).

Zhong et al. [27] examined the association between benzodiazepine and the dementia risk using six observational studies, and explored a potential dose–response pattern. The pooled adjusted risk ratios (RRs) for dementia were 1.49 (95% confidence interval (CI) 1.30–1.72) for long-term users compared with never users. However, the risk of dementia was increased among recent (RR 1.55, 95% CI 1.31–1.83) and past users (RR 1.55, 95% CI 1.17–2.03). The pooled adjusted RRs were almost similar when they calculated effect size after adjusting with anxiety and depression. The risk of dementia among benzodiazepine users increased by 22% for a 20 defined daily dose per year increment (RR 1.22, 95% CI 1.18–1.25), and this finding showed no evidence of heterogeneity (*p* = 0.32; I^2^ = 0.0%).

Islam et al. [9] included 10 observational studies (case-control and cohort design) to evaluate the relationship between benzodiazepine use and dementia risk. A significantly increased dementia risk (OR, 1.78 (95% CI 1.33–2.38) was observed among benzodiazepine users as compared to benzodiazepine non-users. The risk of dementia was almost similar in the short half-life (≤20 h) and long half-life benzodiazepine users. Moreover, benzodiazepine use also significantly increased dementia risk when the effect sizes were adjusted with insomnia, anxiety, depression, and psychiatry.

Lucchetta et al. [25] conducted a systematic review and meta-analysis of 11 observational studies, including 980,860 adults/elderly individuals. Of 11 studies, eight studies reported an increase risk among benzodiazepine users, two studies found no association between benzodiazepine and dementia, and one study reported a protective effect. The overall pooled effect showed a significant association between benzodiazepine use and the risk of dementia (OR 1.38, 95% CI: 1.07–1.77). The risk of dementia was not significantly different among short and long-acting benzodiazepine users (RR 1.09 vs. 1.24).

Penninkilampi et al. [26] to examined the association between benzodiazepine and the risk of dementia in elderly patients, involving 14 observational studies with 159,090 individual and tried to control protopathic bias. The risk of dementia [odds ratio (OR) 1.39, 95% confidence interval (CI) 1.21–1.59 was significantly high among benzodiazepines users. Moreover, the magnitude of the risk of dementia was even higher among long-acting benzodiazepines users (OR 1.21, 95% CI 0.99–1.49) than short-acting benzodiazepines users (OR 1.13, 95% CI 1.02–1.26).

He et al. [28] conducted a meta-analysis to determine the association between the long-term usage of benzodiazepine and the risk of dementia. Patients who were taking benzodiazepine had higher risk of dementia (RR 1.51, 95% CI: 1.17–1.95) compared to non-benzodiazepine users. The pooled RR for developing dementia was 1.16 (95% CI = 0.95–1.41, *p* = 0.150) for longer half-life benzodiazepine and 1.21 (95% CI = 1.04–1.40, *p* = 0.016) for a longer time.

### 3.4. Quality of the Included Studies

The AMSTAR-2 tool was to evaluate the quality of five systematic review and meta-analysis (Check Supplementary File). Of five included studies, four studies were low quality and one study was moderate quality. Table 3 shows the quality of included studies.

## 4. Discussion

**Main findings:** This umbrella review of meta-analyses of observational studies provided an updated, comprehensive overview, and critical assessment of the association between benzodiazepines use and the risk of dementia. Among the five included studies, four studies had high heterogeneity and one study showed no heterogeneity. All the included studies had weak evidence; however, the methodological quality was also low.

**Updated evidence:** Recently, several studies also evaluated the risk of dementia among benzodiazepine users. However, the findings of these studies were inconsistent. Grossi et al. [29] conducted a cohort study using data from England. They reported no association between benzodiazepine use and the risk of developing dementia (IRR 1.06, 95% CI: 0.72–1.60). Moreover, Aldaz et al. [30] conducted a large case-control study (case: 15,212 and control: 62,397) using the Spanish database for pharmacoepidemiologic research in primary care (BIFAP) of the Spanish Agency of Medicines and Medical Devices (AEMPS). Benzodiazepine use was not associated with an increased risk of dementia. In fact, there was no significant difference observed between long- and short-acting benzodiazepine use. A retrospective study from the USA evaluated the association between benzodiazepine exposure and the risk of developing dementia using data from 528,006 veterans (aged 65 or older patients without dementia during a 10-year baseline period) [31]. Patients with benzodiazepine were not associated with dementia, the adjusted risk ration for dementia were 1.06 (95% CI: 1.02–1.10), 1.05 (95% CI 1.01–1.09), and 1.05 (95% CI 1.02–1.09) for low, medium, and high benzodiazepine users. Similarly, a prospective study from the Netherlands included 3526 individuals aged between 70 and 78 years without dementia [32]. After two years of follow-up, no association was observed between benzodiazepine use and the risk of dementia.

In contrary, Baek et al. [16] conducted a retrospective cohort study retrieving health insurance claims data from the National Health Insurance Service (NHIS) database of South Korea, where they included benzodiazepine users aged ≥ 55 years with no history of dementia within the previous 5 years. Although, an increased risk of dementia was reported among benzodiazepine users, the findings of the study mentioned that the association was likely due to confounding by indication. Joyce et al. [17] conducted a case-control study using a longitudinal Medicare claim dataset, where benzodiazepine exposure was between approximately 8% and 13%. The risk of dementia was high among patients with benzodiazepine. In fact, the risk of dementia was even greater among patients with higher levels of benzodiazepine exposure (>365 days over a 2-year period). Brieler et al. [1] endeavored to address the prevailing limitations of previous research studies by mitigating confounding variables. The existing body of research remains inconsistent and contentious due to the intertwined associations of both benzodiazepines and dementia with the risk of dementia. Consequently, it remains uncertain whether benzodiazepines, anxiety, or both are exclusively responsible for the increased dementia risk. To tackle this question, a retrospective cohort study was conducted, utilizing eight-year claim databases and implementing the entropy balancing method to mitigate bias. Among the 72,496 patients examined, 4325 were diagnosed with anxiety. However, only a mere 3.6% of these individuals received sustained benzodiazepine prescriptions, and 9.2% experienced dementia incidents. Upon adjusting for confounding factors, the study revealed that both sustained benzodiazepine use (HR 1.28, 95% CI: 1.11–1.47) and an anxiety diagnosis (HR 1.19, 95% CI: 1.06–1.33) were independently associated with incident dementia among patients aged 65–75. Notably, when comparing anxiety disorder with sustained benzodiazepine use to anxiety disorder alone, no significant association with incident dementia was observed (HR 1.18, 95% CI: 0.92–1.51) after thorough confounder control. Despite the observed link between benzodiazepine use and increased dementia risk, the researchers suggested the need for further investigations to ascertain the presence of a dose–response relationship. Such an association could potentially imply that there is a specific threshold of benzodiazepine dosage that heightens the risk of dementia, even among individuals with anxiety disorders. Additionally, future studies should explore whether the relationship between incident dementia and anxiolytic medications varies across different classes of these medications. In a related study, Guo et al. [2] also delved into the connection between cognitive impairment and the use of Z drugs. Their primary focus was on evaluating the risk of cognitive decline associated with exposure to Z drugs among middle-aged and older patients suffering from chronic insomnia. Conducting a case-control study design, they included patients who met the Diagnostic and Statistical Manual of Mental Disorders, 5th edition (DSM-V) diagnostic criteria for chronic insomnia and had a Pittsburgh Sleep Quality Index (PSQI) score > 7. It is worth noting that the categorization of benzodiazepines in this study was based on the World Health Organization’s Anatomical Therapeutic Chemical classification system. To account for potential confounding factors, the researchers incorporated various laboratory variables such as fasting blood glucose (mmol/L), triglyceride (mmol/L), total cholesterol (mmol/L), low-density lipoprotein cholesterol (LDL-C, mmol/L), high-density lipoprotein cholesterol (HDL-C, mmol/L), serum uric acid (μmol/L), and serum albumin (g/L) levels into their model. The study findings indicated that benzodiazepine exposure density emerged as an independent risk factor for cognitive impairment in middle-aged and older patients grappling with chronic insomnia. However, no discernible correlation was established between the use of Z drugs and cognitive impairment in this context.

**What are needed to be done and Why:** As the number of aging populations increases, the prevalence of dementia is expected to rise in the near future. According to a previous study, the proportion of people aged 60 years or older is projected to increase from 9% in 2019 to 22% in 2050 worldwide [33]. While several drugs are available to slow the progression of the disease, none can cure it. Hence, it is vital to undertake comprehensive research to prevent dementia as a public health priority. Older adults are more susceptible to dementia due to the use of multiple drugs to treat chronic diseases [34]. Identifying drugs associated with dementia is crucial for dementia prevention, improving quality of life and cognitive health. Long-term use of certain drugs such as anticholinergic, antipsychotics, and antiepileptics has been linked to an increased risk of dementia [35,36]. Recently, public attention has been focused on the association between benzodiazepines and the risk of dementia among older adults. However, mixed reports regarding the effect of long-term use of benzodiazepines on dementia risk exist. Our study found weak evidence of this association. Previous study suggested that benzodiazepines can be considered in the short-term treatment of psychiatric disorders, as a sole or combined treatment [37]. The medium to long-term risk-benefit ratio may generate controversy, considering the limited level of evidence available. Furthermore, there is a lack of comprehensive information regarding the duration, dosage, and specific types of benzodiazepines (both short- and long-acting) and their association with the risk of dementia. Therefore, further research is necessary to explore the potential link between benzodiazepines and the risk of dementia, taking into account varying doses and durations. In addition to several protopathic bias, the heterogeneity due to methodological differences among the primary studies may challenge the demonstration of a causal relationship.

It is important to note that an association between benzodiazepines and dementia does not imply causation. Future studies should also examine the risk of dementia in older adults who are prescribed benzodiazepines to alleviate symptoms related to depression, anxiety, and insomnia, especially in the early stages of cognitive decline, as these symptoms have been observed to be connected to early-stage dementia. Additionally, there is a need for biological studies to gain a better understanding of the causal relationship between benzodiazepine use and the increased risk of dementia in older adults.

**Strengths and Limitations:** Our study has both strengths and limitations. One strength is that we included all available meta-analyses to provide an updated and comprehensive analysis of the evidence on benzodiazepines and dementia risk. Additionally, we used AMSTAR-2 to evaluate the methodological quality of the included studies and assessed the strength of the evidence. However, our study also has some limitations that should be addressed. For example, we only included five meta-analyses, and we did not include recently published articles or re-analyze existing data to provide the most up-to-date evidence. Furthermore, we were unable to investigate the association between dose and types of benzodiazepines and the incidence of dementia, as this was beyond the scope of our study. Finally, due to data constraints, we were unable to stratify the impact of benzodiazepines by factors such as age, gender, dosage, or treatment duration, all of which are crucial parameters for evaluating the true nature of the association.

**Recommendation on benzodiazepines use in clinical practice**: While benzodiazepines exhibit varying effects on dementia, they demonstrate effectiveness in addressing several conditions, such as depression, anxiety, and insomnia. Although the evidence from the studies examined in this review is somewhat limited, it is advisable to employ benzodiazepines judiciously to minimize the potential risks associated with long-term usage linked to dementia, rather than discontinuing their usage altogether. Physicians should start treatment with the lowest feasible dose of benzodiazepines for a specified treatment duration. In cases of severe anxiety or insomnia, consideration may be given to employing a higher dose with an extended duration [38,39,40].

Aging plays a significant role in the onset of dementia, and given that benzodiazepines have been associated with cognitive impairment, mobility challenges, neuronal cell apoptosis, and impaired driving skills in older individuals, the short-term use of benzodiazepines with short half-lives is generally regarded as safe and recommended for older adults [41,42]. Since prior studies have not provided conclusive evidence that short- to intermediate-term use of benzodiazepines increases the risk of dementia, physicians should explore alternative pharmacological and behavioral approaches before resorting to benzodiazepines and carefully assess the potential risks and benefits of their use.

## 5. Conclusions

This study examined the associations between benzodiazepine use and the risk of dementia. Although all meta-analyses found significant relationships between benzodiazepine use and dementia risk, the strength of the evidence was weak due to the low methodological quality of the included studies. Therefore, researchers and clinicians should interpret these findings with caution. Future studies should include recent articles, evaluate potential biases, and provide updated evidence that accounts for factors such as dose, duration, and types of benzodiazepines.

## Figures and Tables

**Table 1:** Shows the clinical characteristic of included studies.

Author, Year	Study Design	Search Strategy	Objective	Data Inclusion Period	Publication Bias	Findings
Zhong et al. 2015 [27]	S & M	PubMed, EMBASE, Cochrane library	Quantify the association, and to explore a potential dose–response pattern.	2002–2013	Begg’s and Egger’s test (all *p* > 0.05)	Long-term benzodiazepine use is associated with an increased risk of dementia
Islam et al. 2016 [9]	S & M	MEDLINE, EMBASE, CENTRAL	Evaluate the association between benzodiazepine use and dementia risk	2002–2016	Egger’s test (*p* < 0.05)	Benzodiazepine use is significantly associated with dementia risk
Lucchetta et al. 2018 [25]	S & M	PubMed, LILACS, CENTRAL	Identify whether an association exists between the use of benzodiazepines and the development of dementia.	2011–2017	GRADE rating system	there is an association between BZD use and the development of dementia
Penninkilampi et al. 2018 [26]	S & M	PubMed, MEDLINE, EMBASE, CINAHL, LILACS, CENTRAL	to investigate the risk of dementia associated with the use of benzodiazepines in elderly patients	2002–2017	NR	the association between benzodiazepine use and dementia incidence is not purely an artefact due to protopathic bias
He et al. 2019 [28]	S & M	PubMed, EMBASE, Cochrane library	to determine the relationship between the long-term usage of BDZs and the risk of dementia	2002–2017	Begg’s (*p* = 0.21) and Egger’s (*p* = 0.49) tests	BDZ significantly increases the risk of dementia in the elderly population

Note: NR: Not reported, S & M: Systematic review and Meta-analysis.

**Table 2:** Summary of the meta-analysis findings by pooling all the observational studies on associations of benzodiazepine use and the risk of dementia.

Author, Year	Included Study	No of Study	No of the Total Participants	Overall Effect Size (Reported)	*p*-Value	Heterogeneity (I^2^)	Latest Study Year	Publication Bias	Evidence
Zhong et al. 2015 [27]	Obs.	6	45,391	1.49 (1.30–1.72)	<0.001	35.1	2014	No	Weak
Islam et al. 2016 [9]	Obs.	8	97,091	1.78 (1.33–2.38)	<0.001	99	2016	Yes	Weak
Lucchetta et al. 2018 [25]	Obs.	11	980,860	1.38 (1.07–1.77)	<0.001	98	2017	No	Weak
Penninkilampi et al. 2018 [26]	Obs.	14	923,632	1.39 (1.21–1.59)	<0.001	97.59	2017	No	Weak
He et al. 2019 [28]	Obs.	10	213,964	1.51 (1.17–1.95)	0.002	97	2017	No	Weak

Note: Obs.: Observational.

**Table 3:** Quality assessments of included meta-analysis using AMSTAR-2 tool.

Author, Year	AMSTAR Items																
	1	2 *	3	4 *	5	6	7 *	8	9 *	10	11 *	12	13 *	14	15 *	16	O.R.
Zhong et al. 2015 [27]	Yes	No	No	PY	Yes	yes	PY	Yes	Yes	No	Yes	Yes	Yes	Yes	Yes	Yes	L
Islam et al. 2016 [9]	Yes	No	No	PY	Yes	Yes	PY	Yes	Yes	No	Yes	Yes	Yes	Yes	Yes	Yes	L
Lucchetta et al. 2018 [25]	Yes	Yes	No	Yes	Yes	Yes	PY	Yes	Yes	No	Yes	Yes	Yes	Yes	Yes	Yes	M
Penninkilampi et al. 2018 [26]	Yes	No	No	PY	Yes	Yes	Yes	Yes	Yes	No	Yes	Yes	Yes	Yes	Yes	Yes	L
He et al. 2019 [28]	Yes	No	No	PY	Yes	Yes	PY	Yes	Yes	No	Yes	Yes	Yes	Yes	Yes	Yes	L

Note: PY: Partial Yes; L: Low; M: Moderate; O.R.: Overall Ratings; *: Critical domain.

## Data Availability

Not applicable.

## Supplementary Materials

The following supporting information can be downloaded at: https://www.mdpi.com/article/10.3390/jpm13101485/s1, Figure S1: Selection Strategy.
